# Resistant and susceptible chicken lines show distinctive responses to Newcastle disease virus infection in the lung transcriptome

**DOI:** 10.1186/s12864-017-4380-4

**Published:** 2017-12-28

**Authors:** Melissa S. Deist, Rodrigo A. Gallardo, David A. Bunn, Jack C. M. Dekkers, Huaijun Zhou, Susan J. Lamont

**Affiliations:** 1 Department of Animal Science, Iowa State University, Ames, IA USA; 2 Department of Population Health and Reproduction, School of Veterinary Medicine, University of California, Davis, CA USA; 3 Department of Animal Science, University of California, Davis, CA USA

**Keywords:** Newcastle disease virus, Lung, Chicken, RNA-seq, WGCNA

## Abstract

**Background:**

Newcastle disease virus (NDV) is a threat to poultry production worldwide. A better understanding of mechanisms of resistance and susceptibility to this virus will improve measures for NDV prevention and control. Males and females from resistant Fayoumi and susceptible Leghorn lines were either challenged with a lentogenic strain of the virus or given a mock infection at 3 weeks of age. The lung transcriptomes generated by RNA-seq were studied using contrasts across the challenged and nonchallenged birds, the two lines, and three time points post-infection, and by using Weighted Gene Co-expression Network Analysis (WGNCA).

**Results:**

Genetic line and sex had a large impact on the lung transcriptome. When contrasting the challenged and nonchallenged birds, few differentially expressed genes (DEG) were identified within each line at 2, 6, and 10 days post infection (dpi), except for the more resistant Fayoumi line at 10 dpi, for which several pathways were activated and inhibited at this time. The interaction of challenge and line at 10 dpi significantly impacted 131 genes (False Discovery Rate (FDR) <0.05), one of which was *PPIB*. Many DEG were identified between the Fayoumi and Leghorns. The number of DEG between the two lines in the challenged birds decreased over time, but increased over time in the nonchallenged birds. The nonchallenged Fayoumis at 10 dpi showed enrichment of immune type cells when compared to 2 dpi, suggesting important immune related development at this age. These changes between 10 and 2 dpi were not identified in the challenged Fayoumis. The energy allocated to host defense may have interrupted normal lung development. WGCNA identified important modules and driver genes within those modules that were associated with traits of interest, several of which had no known associated function.

**Conclusions:**

The lines’ unique response to NDV offers insights into the potential means of their resistance and susceptibility. The lung transcriptome shows a unique response to lentogenic NDV compared to a previous study on the trachea of the same birds. It is important to analyze multiple tissues in order to best understand the chicken’s overall response to NDV challenge and improve strategies to combat this devastating disease.

**Electronic supplementary material:**

The online version of this article (10.1186/s12864-017-4380-4) contains supplementary material, which is available to authorized users.

## Background

Chickens offer a relatively environmentally friendly and healthy source of protein in form of both meat and eggs [1, 2]. From backyard flocks to commercial settings, chicken production is scalable. In smaller flocks, chickens are often scavengers, easing management; however, this setting also increases risk of the chickens coming into contact with pathogens, as biosecurity practices are rare. In commercial settings, pathogens spread rapidly due to close quarters of animals. Disease is a continuous threat to food security and also to human health in the cases of zoonotic pathogens.

One alternative strategy to curb the devastating impacts of disease in chickens is to utilize host genetic variation to facilitate breeding for disease resistance. Disease resistance is often not absolute; here it is defined as the ability of an individual chicken to interfere with the pathogen life cycle [3]. Examples of phenotypes associated with disease resistance include lower pathogen load, higher antibody titer, or less morbidity and/or mortality. Mounting an immune response consumes large amounts of energy that the chicken cannot put towards production traits such as growth and egg laying [4]. Ideally, disease resistant chickens will expend less energy in the face of a disease challenge and have enough energy to continue to grow and lay eggs; this trait is called resilience [3].

Selection experiments on immune related traits have been successful, but often it is not known what genes and pathways are being selected upon. This may lead to improvement in resistance to one disease at the cost of increasing susceptibility to another [5].

Genetic solutions work best in combination with other disease control strategies, such as biosecurity, vaccination, and culling. When one or more of those strategies are not options, more emphasis should be placed on the others. In developing countries, where many flocks are scavenging chickens, vaccination to Newcastle disease (ND) is not feasible. Newcastle disease, caused by Newcastle disease virus (NDV), a negative sense, single stranded, RNA virus, results in a variety of symptoms and can result in mortality rates as high as 80% from highly virulent strains [6]. This virus is therefore a threat to food security. Fortunately, previous studies have shown that genetic differences result in different disease outcomes in response to NDV [7–10].

Two experimental chicken lines, Fayoumi and Leghorn, have been classified as relatively resistant and susceptible, respectively, to NDV [11] and to many other pathogens, including avian influenza virus (AIV), Marek’s disease virus, *Salmonella*, and *Eimeria* [12–15]. These inbred lines were used as a discovery platform to identify genes and pathways that may be associated with resistance to NDV via RNA-seq. Characterization of these two lines’ response to NDV is necessary in order to identify possible explanations for their differing response. Previously, the trachea transcriptome from these two inbred lines was analyzed after challenge with lentogenic NDV [11]. To gain a more holistic interpretation, multiple tissues should be examined.

In this study, the lung was chosen for RNA-seq analysis due to its proximity to the site of infection (ocular/nasal), known immune importance [16], and because lentogenic NDV has been classified as a respiratory disease [17]. Lentogenic viruses replicate at the site of infection and require a trypsin-like protease to cleave the fusion protein, a viral protein required for entry into the host cell [18]. The host innate immune system rapidly produces cytokines, chemokines, and interferons in response to the virus, and triggers the adaptive immune response. Antibodies were detected as early as 4 days post infection in the saliva of Leghorns infected with lentogenic NDV [19]. Also, the lung is home to bronchus-associated lymphoid tissue (BALT), which plays a crucial role in antiviral immunity [20], and respiratory immune responses are induced in the lung [16]. Previous studies have examined the lung transcriptome in response to challenges with laryngotracheitis virus, AIV, and infectious bronchitis virus (IBV) [21–24]. Comparing across studies can help identify genes and pathways of importance to multiple viral pathogens in poultry.

## Methods

This study utilized two inbred lines (inbreeding coefficient = 99.95% [25]), Fayoumi (M 15.2) and Leghorn (GHs 6), from the Iowa State University Poultry Farm (Ames, IA). Originating from Egypt, the Fayoumi has a history of harsh natural selection, while the Leghorn has a history of artificial selection for egg-laying traits. For the past 60 years, the only selection pressure placed on the two lines has been for survival and reproduction.

The methods used in the present study were approved by the Iowa State University IACUC (log number 1-13-7490-G) according to the appropriate animal guidelines [26]. At hatch, Fayoumis and Leghorns were placed into a biosafety level II facility and randomly assigned to one of two treatment groups. At 3 weeks of age the challenged birds (*n* = 49) were inoculated via an ocular nasal route with 200 μL of 10^7^EID_50_ of La Sota NDV, 50 μL into each eye and nostril. The nonchallenged birds (*n* = 40) were given 200 μL of phosphate buffered saline (PBS) via the same route, as a mock infection. The challenged and nonchallenged birds were kept in separate rooms. Four ABSL-2 rooms, two per treatment group, were used to house the birds. There was one floor pen in each room, with 20 to 24 birds per pen.

At 2, 6, and 10 days post infection (dpi), one-third of the birds were euthanized for tissue collection. The lung was removed, briefly diced, and placed in RNAlater (ThermoFisher Scientific, Waltham, MA) for short-term storage. Within a week, the samples were removed from the RNAlater solution and placed into a − 80 °C freezer.

Three main factors were included in this study: treatment (challenged, nonchallenged), line (Fayoumi, Leghorn), and time (2, 6, 10 dpi). The three time points were chosen to enable the observation of mechanisms of resistance during both the innate and adaptive immune response. In most cases, the number of males and females were balanced within each treatment group (Table 1).

**Table 1:** Sample sizes per treatment group used for RNA-seq analysis

Treatment Group	Males	Females
Fayoumi, Nonchallenged, 2 dpi	2	2
Fayoumi, Challenged, 2 dpi	2	2
Fayoumi, Nonchallenged, 6 dpi	3	1
Fayoumi, Challenged, 6 dpi	2	2
Fayoumi, Nonchallenged, 10 dpi	2	2
Fayoumi, Challenged, 10 dpi	2	2
Leghorn, Nonchallenged, 2 dpi	2	1
Leghorn, Challenged, 2 dpi	2	3
Leghorn, Nonchallenged, 6 dpi	2	2
Leghorn, Challenged, 6 dpi	2	2
Leghorn, Nonchallenged, 10 dpi	2	2
Leghorn, Challenged, 10 dpi	2	2

Total RNA was isolated from the lung using an RNAqueous kit (Thermo Fisher Scientific, Waltham, MA). The isolated RNA was then DNase treated using a DNA-free kit (Thermo Fisher Scientific, Waltham, MA). All samples had an RNA quality number greater than 8.0, as measured using the Fragment Analyzer™ Automated CE System (Advanced Analytical Technologies, Inc., Ankeny, IA). A 500-ng input was utilized to construct a cDNA library for each sample, using the high-throughput protocol in the TruSeq RNA sample preparation guide (v2; Illumina, San Diego, CA). The cDNA libraries were validated using the Fragment Analyzer™ Automated CE System (Advanced Analytical Technologies, Inc., Ankeny, IA), and then sequenced on the HiSeq2500 platform (Illumina, San Diego, CA) for 100-bp, single-end reads (DNA Facility, Iowa State University, Ames, IA). The 12 treatment groups were balanced across four lanes of the flow cell and randomly assigned an index. Sequence data can be accessed at the ArrayExpress database at EMBL-EBI (www.ebi.ac.uk/arrayexpress) under accession number E-MTAB-5859.

Analysis of the RNA-seq data was performed in the Discovery Environment of iPlant Collaborative [27], now known as Cyverse. The Illumina TruSeq adapter sequence and the individual multiplexing indexes were removed from each sample using the FASTX Clipper program. Sequences less than 30 bp in length, or that did not have a Phred score greater than or equal to 30 in 80% of all bp were filtered out using FASTX. Remaining high quality reads were input into TopHat2 [28] (default parameters; isoform fraction = 0.10) and aligned to the Gallus_gallus-5.0 (Gal5; GCA_000002315.3) reference genome that was downloaded from the Ensembl genome browser. Read counts for each transcript were generated from HTSeq [29] (mode: intersection-nonempty).

For data visualization, pcaExplorer [30] was used to generate principle component analysis (PCA) plots based on DESeq2 [31] normalized counts, while accounting for line, treatment, dpi, and sex using the dds function and the variance stabilizing transformation (vst). The 500 most variant transcripts were used to calculate the variance associated with each principle component.

The count data were statistically analyzed using the generalized linear model option in edgeR [32], accounting for line, treatment, dpi, and sex, to determine the number of differentially expressed genes (DEG) among the 12 treatment groups. A false discovery rate (FDR) less than 0.05 was used to declare DEG. Contrasts were written to compare the challenged and nonchallenged birds, the Fayoumis and Leghorns, and the different time points. A contrast was also written to determine which transcripts were significantly impacted by a challenge*line interaction at each time and these were input into STRING (version 10.0) [33] for network analysis. In STRING, a medium confidence level (0.400) was utilized and disconnected nodes were removed.

Other programs utilized for analysis of DEG included Ingenuity Pathway Analysis (IPA; Qiagen, Redwood City, CA) and Cell type enrichment (Cten) software [34]. Transcripts with a FDR less than 0.05 and absolute LFC greater than 1 were used to generate z-scores and *p*-values in IPA. For Cten, DEG were converted from their Ensembl transcript ID to their associated gene name using Biomart [35], and then input into Cten.

For co-expression analysis, the Weighted Gene Co-expression Network Analysis (WGCNA) package in R was utilized. WGCNA clusters genes into modules based on expression levels and correlates variation in expression levels in those modules to traits of interest. Transcripts with less than four total counts across all samples were removed prior to normalization with DESeq2 [31] for the WGCNA analysis. A soft threshold of 13 was utilized to generate an adjacency matrix based on co-expression. Minimum module size was set to 30. Modules were then correlated to different traits. For discrete traits, including line, dpi, treatment, and sex, each chicken was given a nominal value of 2, 1, or 0. For the continuous traits, viral load was reported in log copy number and antibody load by sample-to-positive ratio. Viral load was obtained by qPCR of the chicken lachrymal fluid at 2 and 6 dpi, and an ELISA for NDV antibody was used to measure serum antibody levels at 10 dpi, as described previously [11].

Transcript IDs with FDR less than 0.05 were converted to associated gene names using BioMart on Ensembl [35]. For gene ontology (GO) analysis, associated gene names were input into the Generic GO term finder [36]. The chicken genome was selected and default parameters were used to generate significant GO terms associated with a set of genes.

The Fluidigm Biomark system (Fluidigm, South San Francisco, CA) was used to further validate the RNA-seq technology. The isolated RNA used to construct the cDNA libraries was also used as input for Fluidigm Biomark. The genes selected for this experiment were chosen to represent a range of fold changes across three tissues: trachea [11], lung, and Harderian gland (Lamont, personal communication). Primers were created based on the previous reference genome, Gallus_gallus-4.0 (Gal4; GCA_000002315.2), and the sequences for the 35 genes used in the current study were previously published [11]. Fluidigm Biomark HD log_2_ fold change (LFC) was calculated by the −2^-ΔΔCT^ method, utilizing *H6PD* as the housekeeping gene. In order to ensure the correct transcript ID was used for correlation to the Fluidigm data, the primer sequences were blasted against the current reference genome, Gal5. If the primers did not find a perfect match or if the matching transcript ID was not found in the edgeR output (likely removed due to low counts or during normalization process), that gene was excluded from the analysis. In two instances (*MHCI-likeY* and *CD40*), primers matched to two different transcript IDs, so the LFC calculated by edgeR for the two transcripts was summed prior to correlation with the Fluidigm Biomark HD LFC.

## Results

### RNA-seq output summary and lung viral load

Approximately 93% of the nearly 12 million filtered reads per sample mapped to the Gal5 reference genome (Table 2). The transcriptome coverage, percentage of transcripts with at least 1 count, was on average 38.96%. Using Gal5 increased the mapping percentage by 2% but decreased the transcriptome coverage when compared to the previous reference genome (Gal4), which is due to the addition of approximately 20,000 transcripts to the Gal5 reference genome. There appeared to be no major differences in the summary statistics of the RNA-seq output (Table 2) between the treatment groups that could cause biases. The unmapped reads were further analyzed to attempt to detect viral transcripts as previously described [11], but no viral transcripts were detected in the unmapped reads of the lung.

**Table 2:** Summary statistics of RNA-seq output

	Pre-filtered Reads	Filtered Reads	^a^Mapping %
Leghorn	15,068,576	12,506,656	92.81
Fayoumi	13,022,086	10,816,065	92.49
Nonchallenged	14,490,133	12,050,144	92.71
Challenged	13,636,113	11,303,680	92.60
All	14,045,331	11,661,361	92.65

^a^Percentage of filtered reads that mapped uniquely to the Gal5 reference genome

### Principle component analysis shows large impact of line and sex on lung transcriptome

**Fig. 1:**
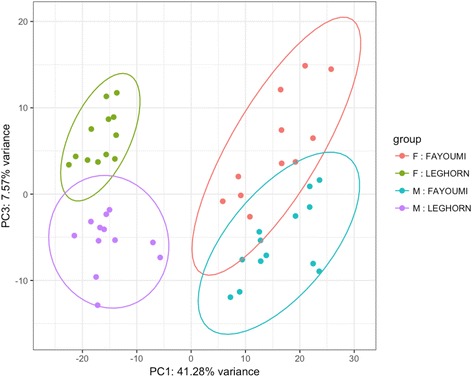
PCA plot suggests genetic line and sex account for large amounts of the variation. pcaExplorer generated principle component analysis (PCA) plot using the top 500 genes to display the variance associated with a principle component (PC). The samples (dots) are color labeled by sex and line. The ellipses around each group are drawn with a confidence interval of 0.95

The PCA plot generated from the 500 genes with the most variance in the lung transcriptome displayed a clear separation by line and sex (Fig. 1). The first principle component (PC1) accounted for 41.28% of the variance in the lung transcriptome and corresponded to the line differences. The second principle component accounted for 13.78% of the variance, but did not correspond to any of design parameters (data not shown). The third principle component (PC3) accounted for 7.57% of the variance in the lung transcriptome and represented sex differences. Due to the large amount of variance accounted for by PC3, sex was incorporated into the edgeR model to determine which genes were differentially expressed (DE). Many genes found in the top and bottom loadings of the PCs are classified as lincRNA or uncharacterized proteins (Table 3). Interestingly, zinc finger protein 366 (*ZNF366*) contributes to the bottom loadings for both PC1 and PC3 and is located on the Z chromosome.

**Table 3:** Top 10 top and bottom loadings for PC1 and PC3

	ID (description)
Top loadings PC1	ENSGALT00000052188 (lincRNA) ENSGALT00000084582 (lincRNA) ENSGALT00000010657 (*SYT8, synaptotagmin 8*) ENSGALT00000085449 (lincRNA) ENSGALT00000074783 (lincRNA) ENSGALT00000084401 (lincRNA) ENSGALT00000082899 (*DPT, dermatopontin*) ENSGALT00000087260 (lincRNA) ENSGALT00000086210 (lincRNA) ENSGALT00000084683 (lincRNA)
Bottom loadings PC1	ENSGALT00000065772 (lincRNA) ENSGALT00000041171 (*CDHR1, cadherin related family member 1*) ENSGALT00000010186 (*LOC428958, Lipase*) ENSGALT00000026579 (*LINC00954, uncharacterized protein*) ENSGALT00000088678 (protein coding) ENSGALT00000023730 (*ANKRD55, ankyrin repeat domain 55*) ENSGALT00000010222 (*LOC424523, uncharacterized protein*) ENSGALT00000012370 (*LOC422316, uncharacterized protein*) ENSGALT00000008605 (*CA4, carbonic anhydrase 4*) ENSGALT00000024201 (*ZNF366, zinc finger protein 366*)
Top loadings PC3	ENSGALT00000067307 (protein coding) ENSGALT00000080873 (*RCJMB04_7i8, uncharacterized protein*) ENSGALT00000046430 (*faf, Female-associated factor FAF*) ENSGALT00000077789 (*Nipped-B homolog-like*) ENSGALT00000086634 (lincRNA) ENSGALT00000054249 (protein coding) ENSGALT00000056168 (protein coding) ENSGALT00000084245 (protein coding) ENSGALT00000085229 (lincRNA) ENSGALT00000050894 (protein coding)
Bottom loadings PC3	ENSGALT00000044087 (*ARRDC3, arrestin domain containing 3*) ENSGALT00000083633 (lincRNA) ENSGALT00000009519 (*IGLL1, uncharacterized protein*) ENSGALT00000024328 (*ACER2, alkaline ceramidase 2*) ENSGALT00000073167 (protein coding) ENSGALT00000024902 (*SLC44A1, solute carrier family 44 member 1*) ENSGALT00000024201 (*ZNF366, zinc finger protein 366*) ENSGALT00000012370 (*LOC422316, uncharacterized protein*) ENSGALT00000018840 (*JCHAIN, joining chain of multimeric IgA and IgM*) ENSGALT00000024747 (*PCGF3, polycomb group ring finger 3*)

### Contrasting challenged and nonchallenged birds within each line and time

The gene expression of challenged and nonchallenged birds was contrasted within each time point and each line. These contrasts resulted in 16 and 101 DEG between the challenged and nonchallenged birds in the Leghorn and Fayoumi, respectively at 2 dpi. Two DEG overlap between the Fayoumi and Leghorn in the contrast of challenged and nonchallenged at 2 dpi: *ZNFX1* and a novel transcript (ENSGALT00000021310). Of the 101 DEG at 2 dpi in the Fayoumis, some genes of interest included *CISH*, *LINGO1*, *TGFBI*, *C6*, and *COL5A1*. The GO term analysis of these 101 genes resulted in no significant immune related GO terms, and most of the top GO terms were related to development, including single organism developmental progress, developmental process, and system development. For the 16 DEG at 2 dpi in the Leghorns, *C1QA*, *MARCO*, and *MPEG1* were immune genes of interest. At 6 dpi, there were no DEG due to treatment in the Fayoumis, but there were 2 DEG in the Leghorns: Metazoa_SRP and unknown. At 10 dpi, there were no DEG due to treatment in the Leghorns. Overall, the limited number of DEG in the Leghorn suggests non-responsiveness to NDV challenge in the lung. In the Fayoumis, 2537 DEG were identified when contrasting the challenged vs. nonchallenged at 10 dpi.

**Fig. 2:**
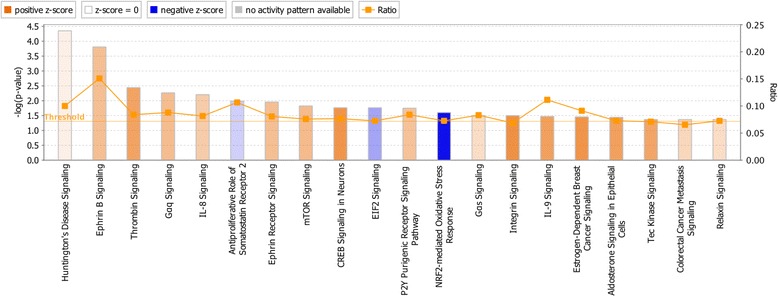
Top canonical pathways in the transcriptome of challenged Fayoumis at 10 dpi. The expression values of transcripts with an absolute log_2_ fold change (LFC) > 1 and a false discovery rate (FDR) < 0.05 were used to calculate the z-scores and –log(*p*-values) for each pathway. Pathways shown in this figure have an absolute z-score > 0.01 and a –log(p-value) > 1.3. Pathways represented are predicted to be either activated (orange) or inhibited (blue). The more intense these colors, the higher the absolute z-score. The height of each bar corresponds to the –log(p-value). The ratio (orange line) represents the proportion of genes within the pathway that were DE

Several pathways were significantly impacted by the challenge at 10 dpi in the Fayoumis, as predicted by Ingenuity Pathway Analysis (IPA; Qiagen, Redwood City, CA) (Fig. 2). Overall, many of these pathways lead to cytoskeleton regulation and cell proliferation/death/movement. In particular, gene expression in the Ephrin B Signaling pathway was predicted to activate cell proliferation, dendrite remodeling, cytoskeleton regulation, and development of focal adhesions. Thrombin signaling was predicted to activate protein synthesis, cell survival, and platelet aggregation. Expression levels in the IL-8 Signaling pathway result in the predicted activation of mobilization of Ca2^+^, exocytosis, endothelial cell migration, endothelial tube formation, angiogenesis, inflammation, cell adherence, neutrophil degranulation, superoxide production, adherence of neutrophils and monocytes, and inhibition of endothelial cell retraction. mTOR signaling results in activated autophagy regulation and actin organization and inhibition of translation. EIF2 Signaling was inhibited, however this pathway was more activated in the nonchallenged Fayoumis than the Leghorns at 10 dpi (data not shown).

### Contrasting the Fayoumis and Leghorns within each treatment group and time

**Fig. 3:**
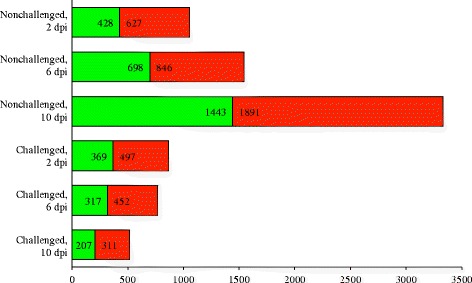
Differentially expressed genes between the Fayoumi and Leghorn at each time within each challenge state. The number of differentially expressed genes (FDR < 0.05) between the Fayoumi and Leghorns are shown for the nonchallenged and challenged birds at each time point. Genes in green were more highly expressed in the Leghorns. Genes in red were more highly expressed in the Fayoumis. The number of genes more highly expressed in each line is labeled within the green and red bar, for each contrast

Although there were relatively few differences between the challenged and nonchallenged birds (with the exception of Fayoumis at 10 dpi), we do see evidence of impact of the challenge on gene expression in the lung when the lines were directly compared (Fig. 3). There were 233 genes consistently DE between Fayoumi and Leghorn regardless of time or challenge, and all of these agreed in LFC direction across contrasts. A total of 441, 512, and 336 genes were shared by the challenged and nonchallenged birds at 2, 6, and 10 dpi, respectively. Of these overlapping genes (DE in both challenged and nonchallenged, Fayoumi vs. Leghorn at each time), all were in agreement in LFC direction, with one exception: at 10 dpi, ENSGALT00000062549 was more highly expressed in the challenged Leghorns, but more highly expressed in the nonchallenged Fayoumis. This novel lincRNA, was also significantly impacted by the challenge*line interaction at 10 dpi.

### The interaction between challenge and line at 10 dpi

**Fig. 4:**
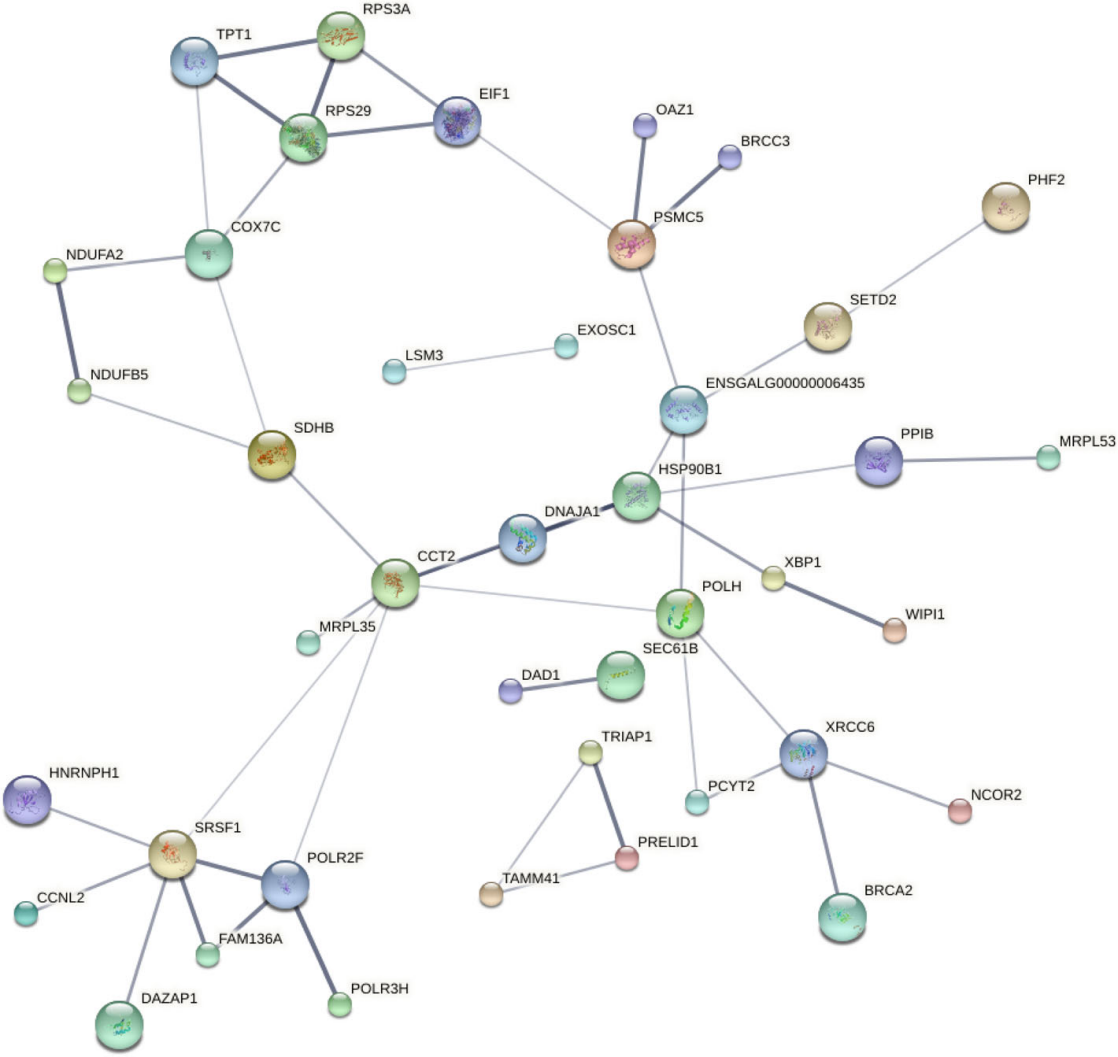
STRING network for genes impacted by the interaction between challenge and line at 10 dpi. Differentially expressed genes (FDR < 0.05) from the challenge*line interaction at 10 dpi were input into STRING for network analysis. Disconnected nodes were removed and a medium confidence score (0.400) was utilized. All edges represent protein-protein interactions; the thicker the edge the more evidence to support that connection. Proteins of unknown structure (small nodes) and predicted structure (large nodes) are represented in various colors

At 2 dpi, only one gene was significantly impacted by both challenge and line at 2 dpi (*NOV-201*), and no genes were impacted by the interaction at 6 dpi. The 131 genes that were significantly impacted by both challenge and line at 10 dpi were further analyzed using STRING network analysis (Fig. 4). A total of 83 nodes and 48 edges were incorporated, which resulted in an average node degree of 1.16 and average local clustering coefficient of 0.305. This network had significantly more interactions than expected (*p* = 4.09e-06) and was significantly associated with the KEGG pathway, protein processing in endoplasmic reticulum (FDR = 0.00838). The genes in this network are highly involved in protein translation and alternative splicing and may be important in antibody production.

### Temporal differences in the lung of nonchallenged Fayoumis and Leghorns

Days post infection appeared to impact the number of DEG, as there were fewer DEG between challenged Fayoumis and Leghorns as time progressed, but there were more DEG between the nonchallenged Fayoumis and Leghorns as time progressed (Fig. 3). The increase in DEG over time in the nonchallenged birds could be related to developmental changes that differ between lines.

**Fig. 5:**
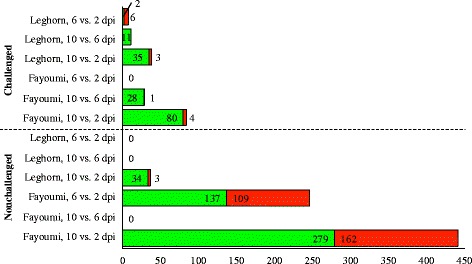
Temporal changes in gene expression within line and treatment group as measured by DEG. Each contrast (y-axis) compares two time points (2, 6, or 10 dpi), within each line (Fayoumi or Leghorn), and within each treatment group (challenged or nonchallenged). The dashed horizontal line separates the challenged birds (top) and nonchallenged birds (bottom). Transcripts more highly expressed in the earlier time point are green and those more highly expressed in the later time point are in red. Numbers within the bar chart correspond to the number of differentially expressed genes

Temporal changes in the lung transcriptome are evidenced by high numbers of DEG when the time points were compared within line and treatment group (Fig. 5). Overall, the nonchallenged Fayoumis showed the most changes over time in numbers of DEG. There were 246 DEG between the nonchallenged Fayoumis at 6 and 2 dpi and 441 DEG between 10 and 2 dpi. Only 145 of the DEG were shared between 6 and 2 dpi and 10 and 2 dpi, suggesting a complex developmental progression. When considering all DEG across time in the nonchallenged Fayoumis, several were immune related. Top GO terms associated with the DEG between 10 and 2 dpi in the nonchallenged Fayoumi included system development, neutrophil chemotaxis, animal organ development, and leukocyte migration, suggesting a close link between developmental changes and immune related changes in the Fayoumi lung over time.

**Fig. 6:**
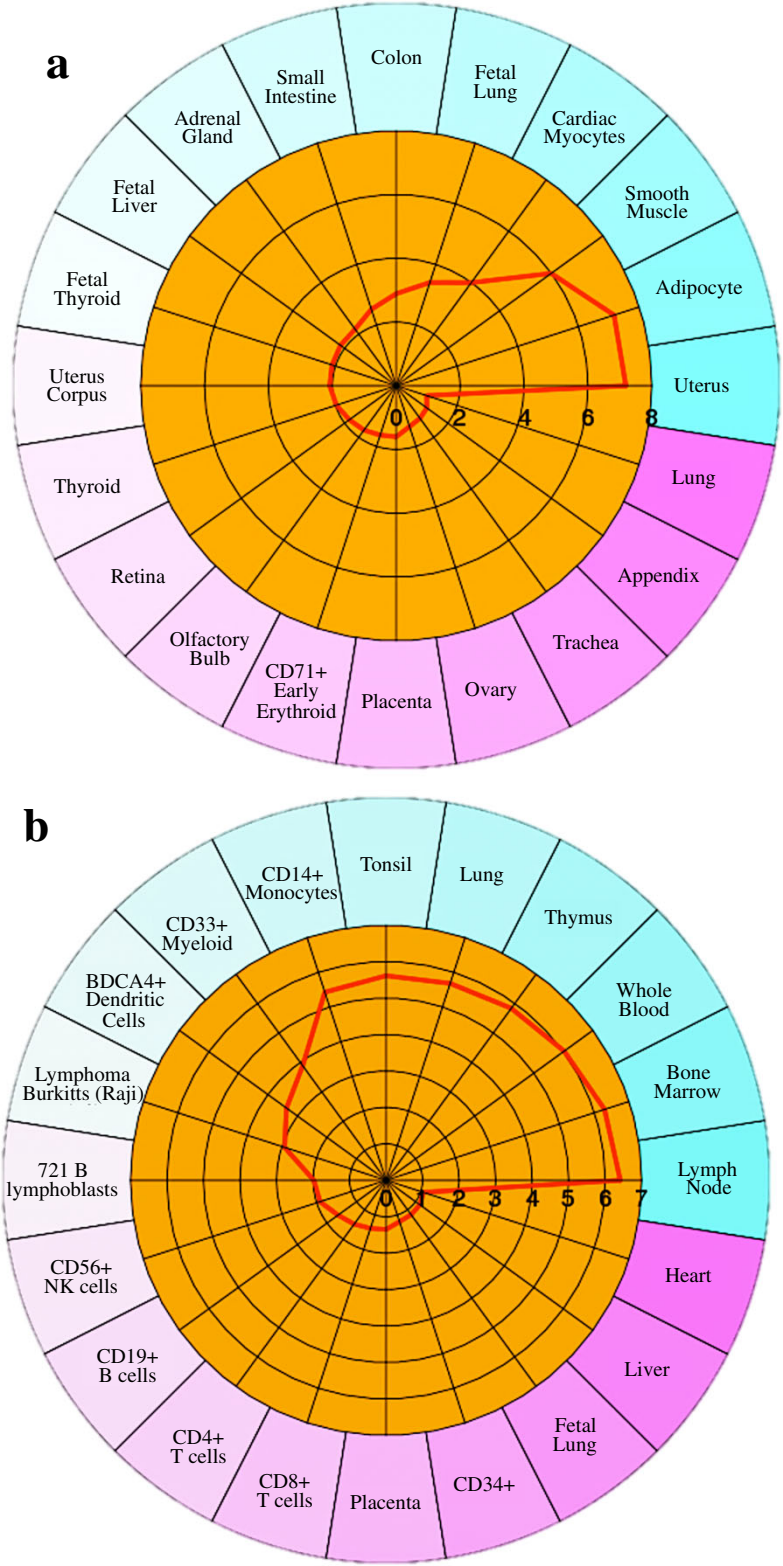
Comparing enriched cell types at 2 and 10 dpi of the nonchallenged Fayoumis. Tissue types (labeled outside circle) with an enrichment score (red line) greater than 2 were considered significant. **a** The transcripts more highly expressed at 2 dpi in the nonchallenged Fayoumis were converted to their associated gene name and input into Cten. Of the 279 differentially expressed transcripts, 187 had an associated gene name, and Cten recognized 126. **b** The transcripts more highly expressed at 10 dpi in the nonchallenged Fayoumis were converted to their associated gene name and input into Cten. Of the 162 differentially expressed transcripts, 95 had an associated gene name, and Cten recognized 69

Changes in gene expression over time may be related to different composition of cells present in the lung. Cell type enrichment (Cten) software [34] predicts enriched cell types based on DEG. The genes that were more highly expressed at 2 and more highly expressed at 10 dpi in the nonchallenged Fayoumis were input into Cten separately for cell type enrichment analysis (Fig. 6). At 2 dpi, a variety of cell types were significantly enriched, including uterus, adipocyte, smooth muscle, cardiac myocytes, fetal lung, and more (Fig. 6a). At 10 dpi, nearly all significantly enriched cell types were immune related, such as lymph node, bone marrow, whole blood, thymus, lung, tonsil, and several specific immune cell types (Fig. 6b). Although chickens do not have lymph nodes, this result may suggest an increase in the bronchus-associated lymphoid tissue of the lung. Between 23 and 31 days of age, the Fayoumi lung appears to be developing its immune competency by increasing the amount of immune related cell types.

In the nonchallenged Leghorns, only 37 genes were DE between 10 and 2 dpi, and 0 genes were DE between the other time points (Fig. 5). GO term analysis on the 37 DEG between 10 and 2 dpi resulted in 2 significant terms: oxygen transport and gas transport. Temporal differences in gene expression were unique to each line. These differences between lines may account for the increase in DEG between nonchallenged Fayoumis and Leghorns as time progressed.

### Different DEG identified over time in the challenged Fayoumis and Leghorns

In the challenged Fayoumis, there were fewer DEG across time overall and the increase in DEG with time seemed to be delayed when compared to the nonchallenged Fayoumis (Fig. 5). Most genes were more highly expressed at the earlier time point in the challenged Fayoumis.

The number of DEG in the challenged Leghorns appeared to be similar to the nonchallenged Leghorns, but the DEG between 10 and 6 dpi and 6 and 2 dpi in the challenged birds were not differentially expressed in the nonchallenged birds. The Leghorns had similar numbers of DEG between 10 and 2 dpi, in the challenged and nonchallenged birds, 38 and 37, respectively (Fig. 5). However, only 1 DEG was shared between these two contrasts (ENSGALT00000007669). The DEG in the challenged birds may not be related to developmental changes and could be a result of the challenge. Only 22 DEG were shared between the challenged and nonchallenged 10 vs. 2 dpi contrasts in the Fayoumis.

### Gene co-expression analysis

**Fig. 7:**
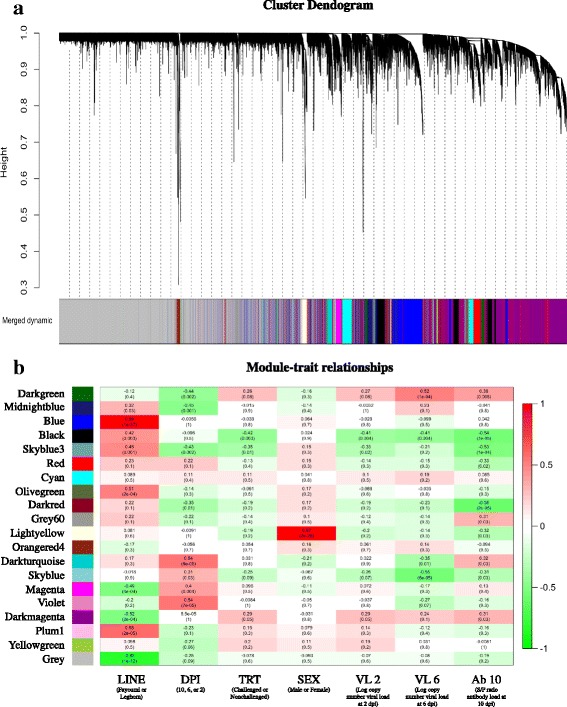
Cluster dendogram and module-trait relationships from WGCNA. **a** Cluster dendogram shows the transcript relationships and their corresponding module. Each color represents a module. **b** Each module (y-axis) is correlated to each phenotype (x-axis) the correlation and p-values were reported for each comparison. Strong positive correlations are colored in red, and strong negative correlations, green. Discrete factors were given a value of 0, 1, or 2. The factor given the higher value is listed first underneath the trait, in parenthesis. The units of the quantitative traits (VL 2, VL 6, and Ab 10) are given underneath the trait in parenthesis. Days post infection (DPI); Treatment (TRT); Viral load (VL); Antibody (Ab)

Due to the large differences in the nonchallenged birds over time and the unexpected numbers of DEG obtained through contrasts over time, WGCNA, which does not rely on contrasts, was utilized. All transcripts and their normalized count values were clustered into modules based on similarities in gene expression (Fig. 7a). Those modules were then correlated with traits of interest (Fig. 7b). GO term analysis was performed to determine the function of genes within modules of interest. Several modules were significantly associated with immune related GO terms. The blue module genes were positively correlated with line, but were not significantly correlated with any other trait measured (dpi, treatment, sex, viral load at 2 and 6 dpi, and antibody levels at 10 dpi). Immune related GO terms that were significantly associated with these genes include defense response, regulation of response to stress, and regulation of inflammatory response. The genes of the black module were positively correlated with line and negatively correlated with treatment, viral load, and antibody levels. Viral life cycle, viral process, cellular response to stress, symbiosis, encompassing mutualism through parasitism, regulation of viral process, and response to stress were all immune related GO terms that were significantly associated with the black module. The skyblue3 module followed a similar pattern in module-trait correlations as the black module, except that skyblue3 module was negatively correlated with dpi and was not associated with viral load at 6 dpi. Genes in the skyblue3 module were significantly associated with integrin-mediated signaling pathway, positive regulation of apoptotic process, positive regulation of programmed cell death, positive regulation of cell death, positive regulation of apoptotic signaling pathway, and response to stress. Genes in the lightyellow module were strongly positively correlated with sex and negatively correlated with antibody levels. Most of the 224 genes in this module were located on the Z chromosome (80%), 4% on the W chromosome, 2% were autosomal, and 14% on scaffolds. The latter may belong to a sex chromosome. Genes within the lightyellow module were also significantly associated with cellular process, metabolic process, and single-organism process.

### Driver genes within modules of interest were examined

Within each module, genes with high absolute gene significance for a trait and high absolute module membership are likely critical hub genes for the module. The top three transcripts, based on highest absolute gene significance, for the module with the strongest correlation for each trait were identified (Table 4).

**Table 4:** Top 3 transcripts for the top modules for each trait

Trait	Module	Top 3 Transcripts (description)	(GS^a^, MM^b^)
Line	Blue	ENSGALT00000052188 (lincRNA) ENSGALT00000087010 (protein coding) ENSGALT00000066798 (lincRNA)	(0.976, 0.964) (0.976, 0.966) (0.974, 0.962)
^c^DPI	Darkturquoise	*RNF208* (Ring finger protein 208) *LAMA2* (Laminin subunit alpha 2) *LCK* (LCK proto-oncogene, Src family tyrosine kinase)	(0.644, 0.753) (−0.644, −0.562) (0.643, 0.74)
Treatment	Black	*cNFI-A* (Nuclear factor 1 C-type) *RHOT2* (Ras homolog family member T2) *UBE2E3* (Ubiquitin conjugating enzyme E2 E3)	(−0.671, 0.694) (0.61, −0.827) (−0.576, 0.812)
Sex	Lightyellow	ENSGALT00000077789 (Nipped-B homolog-like) ENSGALT00000085229 (lincRNA) ENSGALT00000080994 (protein coding)	(−0.987, −0.937) (−0.985, −0.94) (−0.984, −0.964)
2 dpi viral load	Black	*cNFI-A* (Nuclear factor 1 C-type) *RHOT2* (Ras homolog family member T2) *CDC5L* (Cell division cycle 5 like)	(−0.648, 0.694) (0.596, −0.827) (−0.586, 0.681)
6 dpi viral load	Skyblue	*FAM98B* (Family with sequence similarity 98 member B) *FUT8* (Alpha-(1,6)-fucosyltransferase) *ATP6V1H* (V-type proton ATPase subunit H)	(−0.609, 0.777) (−0.605, 0.87) (−0.592, 0.849)
10 dpi antibody level	Darkred	ENSGALT00000068419 (protein coding) *FSTL1* (Follistatin like 1) *NOV* (Nephroblastoma overexpressed)	(−0.754, 0.895) (−0.729, 0.844) (−0.719, 0.794)

^a^Gene Significance (GS)

^b^Module Membership (MM)

^c^Days post infection (DPI)

Many of the top transcripts associated with these traits have limited functional information available, especially functional information specific to chickens. The top transcripts associated with line in the blue module were novel, with no associated gene names. ENSGALT00000052188 was also a top-loading gene for PC1 (Table 3).

Of the transcripts associated with sex, two of the three genes are located on the W chromosome and the other (ENSGALT00000080994) on a scaffold; it is possible this scaffold should be placed on the W chromosome. ENSGALT00000077789 and ENSGALT00000085229 were also top loading genes for PC3 (Table 3). Several novel associations were made between driver genes and traits of interest in this study. Further investigation of the impact of these driver genes on other traits can help elucidate the overall function of the protein.

### Fluidigm biomark was utilized to validate the RNA-seq results

**Fig. 8:**
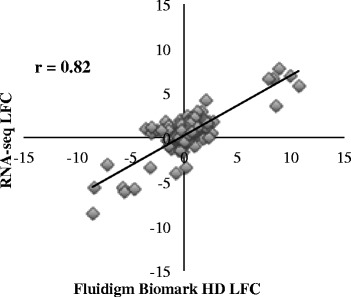
Validation of RNA-seq technology using the Fluidigm HD Biomark system. This plot is composed of 210 points comparing the log_2_ fold change (LFC) as estimated by Fluidigm Biomark HD (x-axis) and RNA-seq (y-axis) for the Fayoumi vs. Leghorn contrasts within each time (2, 6, 10 dpi) and each treatment group (challenged, nonchallenged), resulting in six total contrasts for 35 genes

The RNA-seq results were validated using the Fluidigm Biomark system. Although several genes had low LFC and clustered around zero, the correlation of LFC between the two methods was high (*r* = 0.82) (Fig. 8).

## Discussion

The lung plays a crucial role in the immune system of chickens, especially after challenge with respiratory pathogens. The BALT, located in the lung, is home to B and T cells and can impact the clinical outcome of disease [20]. No detectable virus was found in the lung at the time points measured; however, due to the high virus levels detected in the trachea [11], the respiratory nature of lentogenic NDV [17], and the lung’s importance in local immunity, examining the lung transcriptome was valuable.

### Sex had a large impact on lung transcriptome

There were surprisingly large sex effects in a non-sex related organ in young chickens, likely due to incomplete dosage compensation [37–39]. The lightyellow module, which was strongly positively correlated with sex, included 179 transcripts from the Z chromosome, which is approximately 10% of the total number of transcripts from that chromosome. The overlap of top and bottom loading genes identified in the PCA with driver genes identified from the lightyellow module give strong confidence to the impact of sex on these genes.

Sex differences have previously been related to different disease outcomes in birds [40–42]. Nipped-B homolog-like, ENSGALT00000077789, a driver gene associated with sex in the lightyellow module, was previously associated with hemoglobin levels in mammals [43]. Previous studies have also pointed to the importance of hemoglobin genes in disease resistance to AIV [15]. The lightyellow module was also negatively correlated with antibody levels. Future RNA-seq studies in the chicken should balance and account for sex in their design.

### NDV stimulated a large number of DEG in the Fayoumi at 10 dpi

The large number of NDV-induced DEG at 10 dpi in the Fayoumis strongly suggests unique responses to NDV between the lines. The mTOR signaling pathway was significantly activated in challenged Fayoumis at 10 dpi and based on expression levels was predicted to result in autophagy. Increased autophagy benefits NDV replication [44]. EIF2 signaling, which can lead to inhibition of NDV replication [45], was down regulated in the challenged Fayoumis at 10 dpi, but was more activated in the Fayoumis than Leghorns at 10 dpi in the nonchallenged birds. Previous comparisons have shown this pathway to be more activated in Fayoumi than Leghorn in the trachea and spleen of challenged birds at 2 dpi [11] (Lamont, personal communication). This pathway results in inhibition of viral replication through inhibition of translation and increased apoptosis [45]. Reduced autophagy and increased apoptosis would be beneficial in cells that are infected with the virus, however, NDV was not detectable at any of the time points in the lung transcriptome. The absence of detectable virus in the lungs of these challenged birds alters the strategy used by the host to defend itself from the virus.

Although the specific reason for the relatively low number of DEG between challenged and nonchallenged birds of both lines at the 6 dpi time is not clear, it may relate to a period of transition from innate to acquired immune mechanisms. Immune response as measured by GO term analysis of DEG in the lung was detected as early as 6 dpi in six-week-old birds after challenge with a lowly pathogenic AIV [46]. Different pathogens and ages of the host may cause different response timelines in the lung.

The transcriptomic response in the lung to NDV was unique when compared to the response of the tracheal epithelial cells of the same birds [11]. The number of DEG between challenged and nonchallenged birds decreased over time in the trachea [11]. In the lung the delayed response in the Fayoumi and non-responsiveness in the Leghorn may be due to the lack of detectable virus, while high levels of virus were detected in the trachea of these birds [11]. In contrast, in a separate experiment with commercial Leghorns, 4 days after challenge with AIV a higher viral load was observed in the lung than trachea [47].

Based on gene expression in the lung, 10 dpi appears to be a crucial time in the immune response timeline for the Fayoumis. Genes that were impacted by both challenge and line are of particular interest in the search for genes related to disease resistance. One of these genes is *PPIB,* also known as cyclophilin B, which is a chaperone protein involved in modulating the host immune response [48]. Previous reports have shown that *PPIB* can increase the rate of IgG folding [49–51]. Further investigation into this protein’s role in chickens and in response to NDV is necessary.

A conserved response to this virus by the two lines, as evidenced by the fewer DEG between the challenged Leghorns and Fayoumis as time progressed, may suggest a slow ramp-up of the immune response in the lung. A previous study [15] also found more DEG between nonchallenged Fayoumis and Leghorns than between the AIV challenged Fayoumis and Leghorns in the lung. It is possible that gene expression in the Fayoumi and Leghorn becomes less different after an immune challenge because the two lines are marshaling similar mechanisms of response in the lung. However, these two lines are known to differ in their response to multiple pathogens.

### Genetic selection based on gene expression

The black module genes’ expression levels negatively correlated with viral load and were positively correlated with line. The blue module genes were strongly positively correlated with line, but were not correlated with the other immune traits measured; nonetheless, they were immune related as evidenced by GO term analysis. The basal expression levels of these genes may be important to the relative resistance of the Fayoumi to other pathogens. In practice, it is important not to select on one phenotype or for resistance to one pathogen, as this may increase susceptibility to another pathogen. Selection based on basal gene expression levels has been successful [52]. Genes in the black and blue modules may be good candidates for selection.

### NDV may have impacted normal lung development

Differences between the two lines were also seen in the number of DEG between the multiple time points. A previous study observed an impact of genetic line on lung development in the transcriptomes of mice, which included differences in immune system chemotaxis, neurogenesis, and the extra-cellular matrix composition [53]. The increased DEG seen across time in the nonchallenged Fayoumis compared to the challenged Fayoumis and GO term analyses, unveiled a relationship between the challenge and developmental changes in the lung transcriptome related to the immune system and development. This was likely due to resource allocation.

The fewer DEG between time points in the challenged Fayoumis could be a direct result of the NDV challenge and resource allocation, because their energy was utilized to mount an immune response and, therefore, growth and development were arrested. Of the top driver transcripts associated with traits of interest, *RHOT2*, *ATP6V1H*, ENSGALT00000068419, and *FSTL1* all have associations with weight and growth related traits [54–57]. Repeated appearance of growth related associations among the top driver genes identified by WGCNA provide further evidence that developmental traits may be impacted by NDV challenge.

### WGCNA identified potentially important driver genes

*LAMA2* decreased in expression over time and is associated with collagen and bacterial invasion [58] and important for CD4+ CD8+ immature thymocytes in mice [59]. The relationship between collagen and immune cell development was previously identified as a potential mechanism of resistance to NDV in the Fayoumi [11]. *LCK* increases in expression over time and is known to be involved in T cell receptor signaling. The expression levels of genes within the darkturquoise module were also negatively correlated with viral load at 6 dpi and positively correlated with antibody levels at 10 dpi. Previous studies have shown the importance of cell mediated immunity in host defense against NDV, but cell mediated immunity is not sufficient to protect the host, as antibodies are also important for lasting immunity [60, 61]. These genes are likely critical to the chicken’s overall response to NDV, regardless of genetic background.

## Conclusions

Although no virus was detected in the lung, the reported results are still of value in observing how the lung tissue responds when the animal is infected. Transcriptional profiling in a tissue may reveal a response to events that occur at earlier times in the same tissue, or at remote sites in the body (communicated via circulating cells or molecules). Line and sex each had a large impact on the lung transcriptome as seen by PCA and WGCNA. The two lines responded uniquely to NDV, which offers insights into their mechanisms of resistance and susceptibility. The challenge may have interrupted normal lung development, which was also different between the two lines, as measured by numbers of DEG between the time points. Genes that were significantly impacted by the interaction between challenge and line at 10 dpi, especially *PPIB*, may play a critical role in antibody production. The response of the lung transcriptome was distinctly different from that of the trachea epithelial cells of the same birds [11]. This highlights the value of examining multiple tissues and time points to more comprehensively understand the mechanisms of disease resistance in chicken. Future studies should examine protein expression levels, immunohistochemistry, and gene knockouts and their response to multiple strains of NDV to confirm true mechanisms of resistance.

## Additional files

Additional file 1: Contains the edgeR output from all the contrasts analyzed in this study. Only transcripts with FDR < 0.05 were included. Contrasts with no significant DEG were not included. (XLSX 1106 kb)

Additional file 2: Contains the top and bottom loading transcripts for PC1 and PC3 from pcaExplorer. (XLSX 46 kb)

Additional file 3: Contains the WGCNA output for each trait. For each transcript the module membership scores and *p*-values for all modules were included, and the gene significance score and p-value were given for each trait. (XLSX 81939 kb)

## Abbreviations

Ab: Antibody
AIV: Avian influenza virus
Cten: Cell type enrichment
DE: Differentially expressed
DEG: Differentially expressed genes
dpi: Days post infection
FDR: False discovery rate
Gal4: Gallus_gallus-4.0
Gal5: Gallus_gallus-5.0
GO: Gene ontology
GS: Gene Significance
IBV: Infectious bronchitis virus
IPA: Ingenuity Pathway Analysis
LFC: Log_2_ fold change
MM: Module Membership
ND: Newcastle disease
NDV: Newcastle disease virus
PBS: Phosphate buffered saline
PC: Principle component
PC1: First principle component
PC3: Third principle component
PCA: Principle component analysis
TRT: Treatment
VL: Viral load
WGNCA: Weighted gene co-expression network analysis
